# Development of a UiO-66 Based Waterborne Flame-Retardant Coating for PC/ABS Material

**DOI:** 10.3390/polym16020275

**Published:** 2024-01-19

**Authors:** Shaojun Chen, Youhan Zeng, Weifeng Bi, Haitao Zhuo, Haiqiang Zhong

**Affiliations:** 1 College of Materials Science and Engineering, Shenzhen University, Shenzhen 518053, China; chensj@szu.edu.cn (S.C.); 2110343072@email.szu.edu.cn (Y.Z.); a773122527@163.com (W.B.); 2 College of Chemistry and Environment Engineering, Shenzhen University, Shenzhen 518053, China; 3 Guangdong Provincial Enterprise Key Laboratory of Smart Automotive Display, Varitronix (Heyuan) Display Technology Limited, Heyuan 517000, China

**Keywords:** MOFs, flame retardant polymer, PC/ABS, coating

## Abstract

The flame-retardancy of polymeric materials has garnered great interest. Most of the flame retardants used in copolymers are functionalized additives, which can deteriorate the intrinsic properties of these materials. As a new type of flame retardant, functionalized metal–organic frameworks (MOFs) can be used in surface coatings of polymers. To reduce the flammability, a mixture of phytic acid, multi-wall carbon nanotubes, zirconium-based MOFs, and UiO-66 was coated on a PC/ABS substrate. The structure of the UiO-66-based flame retardant was established by FT-IR, XRD, XPS, and SEM. The flammable properties of coated PC/ABS materials were assessed by LOI, a vertical combustion test, TGA, CCT, and Raman spectroscopy. The presence of a UiO-66-based coating on the PC/ABS surface resulted in a good flame-retardant performance. Heat release and smoke generation were significantly reduced. Importantly, the structure and mechanical properties of PC/ABS were less impacted by the presence of the flame-retardant coating. Hence, this work presents a new strategy for the development of high-performance PC/ABC materials with both excellent flame-retardancy and good mechanical properties.

## 1. Introduction

The fire risk posed by polymeric materials has received considerable attention. The hydrocarbon skeleton and organic composition of these materials make them highly flammable. Heat and toxic gases generated during combustion are a great threat to human beings and the environment [1,2]. Many studies have been conducted to prevent the combustion of polymer materials and thereby reduce the harmful effects of combustion [3,4,5,6,7]. The simplest method is to blend specific flame retardants with the polymer matrix. An intumescent flame-retardant system is commonly used, which obstructs the transfer of heat and oxygen by forming an expansive carbon layer on the polymer surface during thermal degradation of the polymer matrix [8,9,10]. Another flame-retardant system that is used is based on catalytic principles, wherein specific catalysts promote a reduction in volatile fragments and toxic gases during the degradation of the polymer matrix. Although the use of these flame-retardant systems and others have been effective in reducing the flammability of polymer, there is a huge demand for flame retardants having a high efficiency.

A flame-retardant coating on the surface of a substrate represents an important method for controlling flammability. Compared with the method of adding flame retardants, the intrinsic properties of the polymer are not compromised in the surface coating method. This method also has the advantage of easy processing and can be used for a variety of materials [11]. In recent years, metal organic frameworks (MOFs), which are organic–inorganic hybrid materials comprising metal ions and organic ligands, have been found to have flame-retardant properties. At high temperatures, MOFs decompose to form catalytic species that promote oxidation and coking [12,13,14]. In particular, a zirconium organic framework, UiO-66, has been found to be a promising material for use in retardant systems. It has a high specific surface area (1000 m^2^/g) [15] and excellent thermal, water, and acid stability, which is conducive to the post-synthesis modification of UiO-66. However, applying MOFs alone cannot provide sufficient flame-retardancy.

A mixture of materials containing UiO-66 can provide excellent flame-retardancy to the system [16]. As a green biomass material, phytic acid (PA) has a high phosphorus content. It can readily chelate with MOFs to provide a composition with better flame-retardancy. In addition, multi-walled carbon nanotubes (MWCNT) have a high carbon content and excellent thermal properties [17,18,19]. These materials are commonly used as components of high-efficiency green flame retardants. Polycarbonate/acrylonitrile-butadiene-styrene copolymer (PC/ABS) materials are widely used in many products, due to their excellent impact strength. However, the presence of a flame-retardant additive greatly influences the mechanical properties of the blend. In an effort to obtain an excellent flame-retardant PC/ABS material, an aqueous flame-retardant system for coating of a PC/ABS surface was prepared using UiO-66, PA, and MWCNT. The as-prepared UiO-66@PA@MWCNT was carefully characterized by Fourier transform infrared (FT-IR) spectroscopy, X-ray diffractometry (XRD), X-ray photoelectron spectroscopy (XPS), and scanning electron microscopy (SEM). The effectiveness of the UiO-66 flame-retardant coating for PC/ABS was evaluated by the limiting oxygen index (LOI), vertical combustion test, thermogravimetric analysis (TGA), cone calorimeter test (CCT), and Raman spectroscopy.

## 2. Experiments

### 2.1. Materials

Polycarbonate (PC, injection grade, QiMei Company, Taiwan), acrylonitrile-butadiene-styrene copolymer (ABS, injection grade, QiMei Company, Taiwan), zirconium chloride (ZrCl_4_, 98%, Shanghai Aladdin Biochemical Company, Shanghai, China), terephthalic acid (99% TPA, Shandong Yusuo Chemical Technology Co., Ltd., Shandong, China), carboxylated multi-walled carbon nanotubes (95% MWCNT-COOH, Suzhou Tianke Trading Co., Ltd., Suzhou, China), N,N-dimethylformamide (99.5% DMF, Shanghai Aladdin Biochemical Company, Shanghai, China), ethanol (99% EtOH, Shanghai Aladdin Biochemical Company, Shanghai, China), and SiO_2_ (99.5%, Jiangsu Tianxing New Materials Co., Ltd., Changshu, China) were used for the experiments.

### 2.2. Synthesis of UiO-66

ZrCl_4_ (1.98 g) and TPA (1.76 g) were weighed and dissolved in 150 mL of DMF. After ultrasonication for 20 min, 4 mL acetic acid was added and the solution was stirred continuously for 10 min. Thereafter, the reaction mixture was transferred to a blue bottle and placed in a constant-temperature vacuum-drying oven at 120 °C. After 36 h, the bottle was taken out and cooled to room temperature naturally. After centrifugation at 6000 r/min for 15 min, the white precipitate was filtered and washed three times with DMF and ethanol. Finally, the white product was dried in a vacuum oven at 85 °C for 36 h, and UiO-66 was collected for further use.

### 2.3. Synthesis of MWCNT@UiO-66

ZrCl_4_ (1.98 g) and MWCNT-COOH (0.20 g) were weighed and dissolved in a blue bottle containing 150 mL of anhydrous DMF. After dispersion by ultrasonication for 20 min, TPA (1.76 g) and 4 mL of acetic acid was added and ultrasonicated for another 20 min. After 36 h of reaction, the blue bottle was taken out and cooled to room temperature naturally. After centrifugation at high speed of 6000 r/min for 15 min, the black precipitate obtained was filtered out and further centrifuged three times each with DMF and ethanol. The product was placed in a constant-temperature vacuum-drying oven at 85 °C for 36 h. Finally, the MWCNT@UiO-66 was collected and stored.

### 2.4. Synthesis of PA@MWCNT@UiO-66

**Figure 1:**
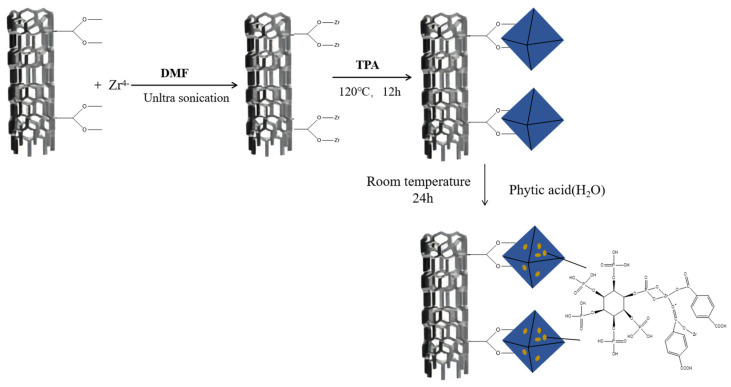
Route for the synthesis of PA@MWCNT@UiO-66.

MWCNT@UiO-66 (1.0 g) was weighed and dispersed in a blue bottle containing 150 mL DMF. Then, PA (10.68 mL) was added to the above MWCNT@UiO-66 suspension, followed by ultrasonic dispersion for 20 min. Thereafter, the mixture was continuously stirred for 24 h at room temperature using a magnetic agitator. The black precipitate obtained was filtered and centrifugally washed with DMF and ethanol 3 times. The final PA@MWCNT@UiO-66 product was then dried in a vacuum-drying oven at 85 °C for 24 h. The synthetic route is presented in Figure 1.

### 2.5. Preparation of Aqueous Flame-Retardant Coatings

For comparison, different MOFs-based flame retardants were evenly dispersed in water-based acrylic emulsion by adding an appropriate amount of water and a small amount of SiO_2_ according to Table 1. SiO_2_ was added as a filler to improve the hardness of coatings and reduce the viscosity. The aqueous emulsions were prepared in a centrifugal tube, by stirring evenly at room temperature using a vortex oscillator, and then dispersed by ultrasonication for 20 min.

### 2.6. Preparation of Flame Resistant PC/ABS

The dried PC/ABS particles were transferred to a polytetrafluoroethylene (PTFE) mold in a plate vulcanization machine, with the temperature set at 240 °C. After heating for 6 min, hot pressing at 18 MPa for 4 min, and cold pressing at room temperature for 2 min, the PC/ABS films were cut into three types of specimens having dimensions of 125 mm × 13 mm × 3 mm, 150 mm × 4 mm × 10 mm, and 100 mm × 100 mm × 3 mm. Some control specimens were reserved for the vertical combustion test, oxygen index test, and cone calorimetry test, whereas the other specimens were sprayed with the aqueous flame retardants, and were placed in a 60 °C electric blast-drying oven for 4 h. The steps for spraying and drying were repeated until the coating thickness reached the standard specification (±10%), as shown in Table 2. The thickness of the coating was determined by measuring the spline thickness before and after coating, using a Vernier caliper.

### 2.7. Characterizations

Fourier transform infrared spectra (FT-IR) were acquired on a Nicolet 6700 infrared spectrometer in reflectance mode. The spectral range was 4000–500 cm^−1^ at a resolution of 4 cm^−1^. The powdered samples were generally measured by the KBr pellet method.

An AXIS Ultra XPS instrument (Shimadzu Company of Japan, Kyoto, Japan) was used for testing the chemical states of elements. Its cathode was made of a lanthanum–aluminum–molybdenum alloy, and the analysis was conducted with a power input of 400 W. The background was a non-rotating background compensation type, and the peak function was the Lorentz–Gaussian function. The powder to be tested was adhered to the tin foil with viscose, folded and pressed, then unfolded and cut into specimen of 5 mm × 4 mm. Chemical states, such as C1s, Zr3d, P2p, and O1s, were determined by scanning the surface of the sample.

The morphologies and elemental distributions of the samples were studied by scanning electron microscopy (NGB4-DXS-10AC, Nanjing Grand Technology Co., Ltd., Nanjing, China). The section of the block sample to be observed was made brittle in liquid nitrogen, and the powder sample and block sample were spray coated with gold for 75 s, after which they were attached to the sample table with conductive adhesive for observation.

TGA and DTG analyses of the samples were conducted using a TGA 55 instrument in a nitrogen atmosphere at a 50 mL/min flow rate. Except when specified, the heating rate was 10 °C/min, and the temperature range was from room temperature to 800 °C.

An X-ray diffractometer (SmartLab, Boston, MA, USA) was used to characterize the material phases in order to analyze their internal structure and morphology. The wavelength was 1.5406 A and the scanning speed was 10°/min in the scanning range of 5–70°.

Vertical combustion tests were performed on uncoated and coated samples according to the GB/T2408-2008 combustion test standard [20]. Each sample was tested thrice and the average value of three readings was used to determine its corresponding flame-retardant level.

The limiting oxygen index (LOI) was expressed according to the percentage of oxygen in the volume. A JF-3 oxygen index instrument was used for the LOI test. The specimen dimensions were 150 mm × 4 mm × 10 mm, according to the standard GB/T2406.2-2009 test [21].

The British FTT cone calorimeter was used for the cone calorimetry test (CCT), according to the ISO5660-1 and ASTM D7309 test standard [22]. The irradiation power was 50 kW/m^2^, sample dimensions were 100 mm × 100 mm × 3 mm, and three parallel tests were carried out for each sample.

The structure, morphology, and graphitization degree of the carbon layer were characterized by FT-IR, SEM, and Raman spectroscopy. The Raman spectra of the residual carbon residue after combustion by CCT were acquired using an excitation wavelength of 514.5 nm. Based on the above results, the mode of action of flame-retardant materials was clarified.

## 3. Results and Discussion

### 3.1. Structural Analysis of Flame-Retardant UiO-66@PA@MWCNT

**Figure 2:**
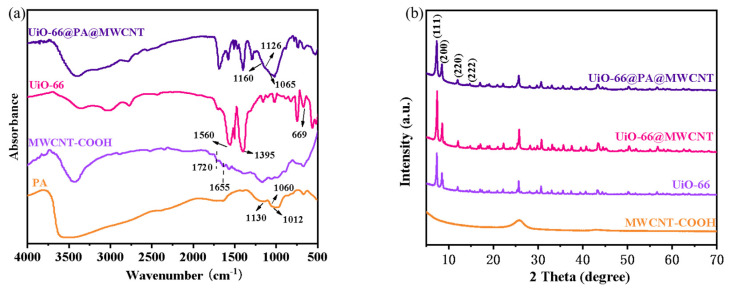
FT-IR spectra (**a**) and XRD patterns (**b**) of UiO-66@PA@MWCNT, UiO-66@MWCNT, MWCNT−COOH, and UiO-66.

First, the zirconium organic framework, UiO-66, was synthesized using ZrCl_4_ and TPA. The obtained UiO-66 was then functionalized with MWCNT and PA to obtain MOFs, labeled as UiO-66@PA@MWCNT. The structure was carefully characterized by FT-IR, XRD, XPS, and TG-DTG during the process. FT-IR spectroscopy was used to determine the molecular structure of UiO-66@PA@MWCNT. In Figure 2a, strong absorption peaks appeared at 1560 cm^−1^ and 1395 cm^−1^ in the FT-IR spectrum of UiO-66. They corresponded to the O-C-O asymmetrical and symmetrical stretching vibrations of TPA, which served as ligands for MOFs. The absorption peaks at 1507 cm^−1^ corresponded to the C=C of the benzene ring, while the peak at 669 cm^−1^ was consistent with the asymmetric stretching vibrations of Zr-(OC). In MWCNT-COOH, the peak for C=O stretching vibrations appeared at 1720 cm^−1^. The small peak at 1655 cm^−1^ was due to the O-H bending vibrations. This confirmed that the carboxyl group was successfully grafted onto MWCNT-COOH, suggesting a good compatibility with PA and UiO-66. The FT-IR spectrum of PA also showed some typical peaks of phosphate groups, including P=O (1130 cm^−1^), p-O-C (1060 cm^−1^), and P-O (1012 cm^−1^). The high phosphorus content can provide a synergistic flame-retardant effect in UiO-66@PA@MWCNT. After MWCNT-COOH, PA, and UiO-66 were reacted, several absorption peaks of PA shifted to higher wave numbers in UiO-66@PA@MWCNT. These results indicated that there was a complex formed between the P-O bond and metal zirconium from UiO-66, verifying the successful synthesis of UiO-66@PA@MWCNT. The XRD patterns in Figure 2b show that the diffraction peaks of UiO-66 were basically consistent with that of the standard profile. After modification with PA and MWCNT, the peak pattern of UiO-66 remained almost unchanged. This indicated that the crystal structure of UiO-66 remained intact during the preparation of functionalized UiO-66.

**Figure 3:**
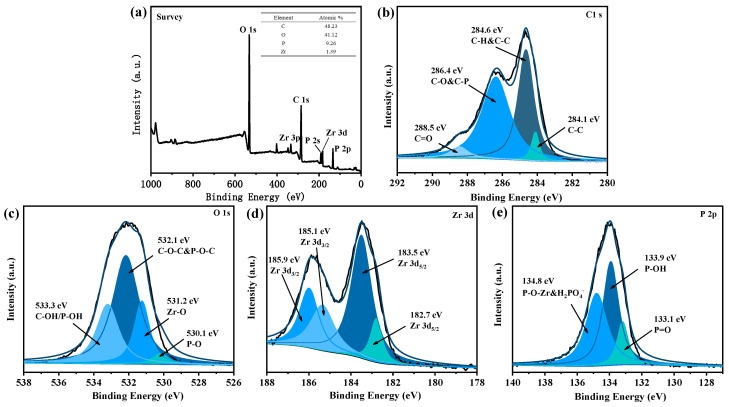
XPS spectra of UiO-66@PA@MWCNT: (**a**)survey scan; (**b**) C1s; (**c**) O1s; (**d**) Zr3d; (**e**) P_2p_.

XPS was used to detect the elements and bonding nature in UiO-66@PA@MWCNT. Firstly, the overall spectrum in Figure 3a showed that the obtained powders were rich in C, O, Zr, and P. Secondly, the energy spectrum of C1s is presented in Figure 3b. After deconvolution of the peaks, it was found that the peaks at 284.1 eV and 284.6 eV corresponded to the neutral bond and sp2-hexagonal network structure of UiO-66@PA@MWCNT, respectively. The peak at 286.4 eV was due to the carbon atom of the C-O bond from UiO-66 and the C-P bond from PA. The peak for C=O at 288.5 eV was consistent with the carboxyl group of the ligand. Thirdly, the energy spectrum of O1s in Figure 3c can be deconvoluted into four peaks. Among them, the peak at 530.1 eV corresponded to the P-O bond of PO_4_^3−^ and HPO_4_^2−^ of PA, while the peak at 531.2 eV was consistent with the Zr-O bond. The peaks at 532.1 eV and 533.3 eV corresponded to C-O-C and P-O-C and C-OH and P-OH, respectively. Fourthly, Figure 3d also shows that the Zr3d spectrum consisted of two peaks, which were further deconvoluted into four peaks. The peaks at 185.1 eV and 185.9 eV corresponded to Zr3d3/2, while the peaks at 182.7 eV and 183.5 eV corresponded to Zr3d5/2. Finally, the P2p spectrum in Figure 3e was composed of three peaks at 133.1 eV (P=O), 133.9 eV (P-OH), and 134.8 eV (P-O-ZR and HPO_4_^2−^). Additionally, TGA and DTG curves (see Supporting Information Figure S1) showed that the carbon residue percentage at 800 °C of UiO-66@PA@MWCNT was 61.3 wt%, which was much higher than that of pure UiO-66. It implied that PA and MWCNT promoted carbon formation in UiO-66@PA@MWCNT. The SEM image of UiO-66 showed a dispersed and smooth octahedral nanocrystal structure (see Supporting Information Figure S2). The reason is that the carboxyl groups on the surface of MWCNT provided sites for the growth of UiO-66, which reduced the stacking of carbon tubes during the in situ growth of UiO-66 along the carbon tubes. The steric hindrance of the carbon tube limited the crystal size of UiO-66, and the grain refinement guaranteed the uniformity of film coating on the substrate by water-based flame-retardant coatings. Hence, these results confirmed the synthesis of functionalized MOFs UiO-66@PA@MWCNT.

### 3.2. Structure Analysis of Flame Resistant PC/ABS Coating

**Figure 4:**
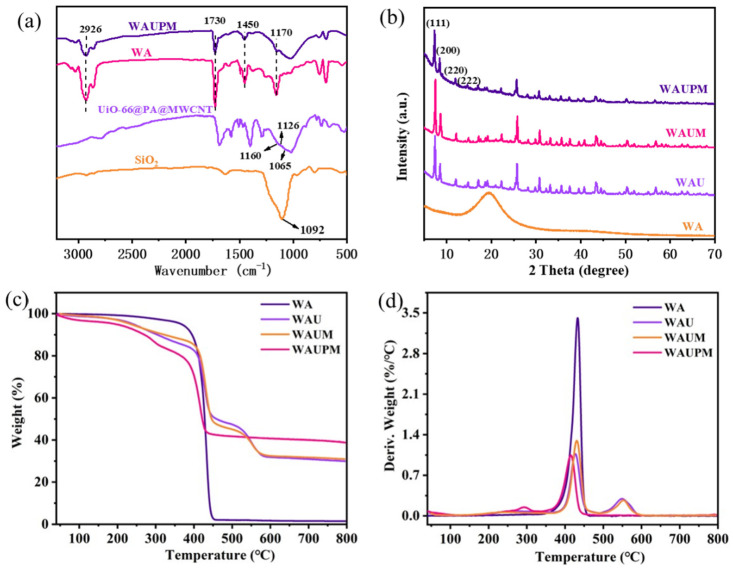
FT-IR spectra (**a**) and XRD patterns (**b**) of WAUPM, WA, UiO-66@PA@MWCNT and TGA (**c**) and DTG (**d**) curves of WAUPM, WAUM, WAU, and WA.

To improve surface adhesivity, UiO-66@PA@MWCNT was dispersed in a water-based acrylic emulsion. The MOFs-based acrylic emulsion was characterized carefully before use. For characterization, films of the MOFs-based acrylic resin were prepared by drying the emulsion in the oven at 85 °C. Figure 4 presents the FT-IR spectra, XRD patterns, and TG-DTG curves of the films. In the FT-IR spectra, the peaks at 1170 cm^−1^, 1450 cm^−1^, 1730 cm^−1^, and 2926 cm^−1^ for the WAUPM sample were attributed to C-O-C stretching vibrations, -OH bending vibrations, C=O stretching vibrations, and methylene stretching vibrations of the acrylic resin, respectively. Additionally, the vibrations for P=O (1160 cm^−1^), P-O-C (1126 cm^−1^), P-O (1065 cm^−1^), Si-O-Si (1092 cm^−1^), and O-C-O symmetric vibrations (1405 cm^−1^) and asymmetric vibrations (1560 cm^−1^) of the terylene ligand were also observed for the WAUPM sample. Comparison with the FT-IR spectra of raw materials, viz. WA, PA@MWCNT@UiO-66, and SiO_2_, showed no deviation of peaks for the WAUPM sample. This implied that the flame retardant did not react with the acrylic resin, which was added as an adhesive.

Furthermore, the XRD spectra showed diffraction peaks consistent with those of UiO-66 for the samples WAM, WAUM, and WAUPM [23]. This indicated that the crystal structure of UiO-66 did not change after functionalization. The TGA curve of WA indicated a good thermal stability, since the weight remained constant until around 443.1 °C. After the addition of UiO-66 or MWCNT@UiO-66, the initial degradation temperature decreased, and a secondary weight loss stage occurred at 550 °C, due to the collapse of the framework structure. WAU and WAUM showed more than 30.9 wt% carbon residue below 800 °C. After further modification with PA, the thermal stability decreased in the initial stage, but the residual carbon rate increased to 38.8 wt% below 800 °C for WAUPM. This result indicated that PA further promoted the char formation process during pyrolysis and also provided a rich carbon source to flame retardants.

**Figure 5:**
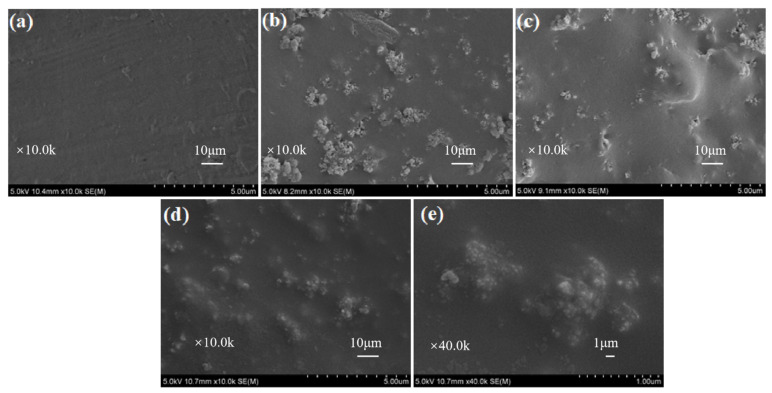
SEM images of untreated PC/ABS (**a**), WAU-3 (**b**), WAUM-3 (**c**), and WAUPM-3 (**d**,**e**).

Different from the additive flame retardants, the PC/ABS flame retardants with different coating thicknesses were prepared by spraying the MOFs-based acrylic emulsions on the surface of PC/ABS. The steps of spraying and drying were repeated until the coating thickness reached the required standard (±10%). The micro-structures of the coated PC/ABS substrates were studied by SEM. As shown in Figure 5, the untreated PC/ABS showed a slightly but uniformly fluctuating surface, while the PC/ABS coated with a MOFs-based flame retardant showed many granular structures on the substrate, presumed to be UiO-66 crystals. It was also found that the substrate became smoother when the thickness of the flame-retardant coating was increased. As seen from Figure 5c,d, the PC/ABS substrates were coated completely above a 750 μm thickness. There were less prominent granular structures on the WAUM-3 surface. WAUPM-3 showed a choppy surface morphology, but with fewer bright MOFs and smoother surfaces. In the magnified SEM images, smaller SiO_2_ nano-particles and larger MOFs were observed, as shown in Figure 5e. Compared with WAUM-3, WAUPM-3 showed an improved surface smoothness. It implied that PA improved the compatibility of MWCNT@UiO-66 with acrylic resin. It also improved the wrapping and maximized the interfacial bonding between the adhesive layer and the PC/ABS substrate. Thus, PA@MWCNT@UiO-66 can be expected to provide a good flame-retardancy to PC/ABS.

### 3.3. Performances of Flame Retardant

**Figure 6:**
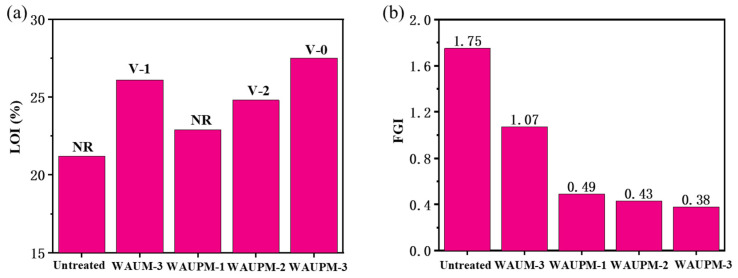
Limiting oxygen index and vertical burning grade (**a**) and fire growth index (FGI) (**b**) of PC/ABS samples coated with a waterborne flame-retardant coating.

Samples with different thicknesses of coating (250 μm, 500 μm, and 750 μm) were dried to test the flame-retardant performance, and the results are presented in Figure 6a and Supplement Material Table S2. As the coating thickness increased, the LOI values of WAUPM-1, -2, and -3 samples gradually increased (22.9%, 24.8%, and 27.5%). WAUPM-3 achieved the UL-94 V-0 grade, while WAUM-3 with the same thickness achieved a V-1 grade. The decrease in flame-retardancy was due to the absence of phosphorus source from PA. The flame-retardancy of the WAU-3 sample was poor and it failed to pass the V-2 grade. This was because this coating easily fell off during the vertical combustion test, reflecting weak adhesion, which was consistent with SEM analysis. Overall, WAUPM-3 achieved ideal flame-retardancy through the coordination of phosphate–zirconium–carbon flame-retardant elements. As shown in Table S2, the total of the two combustion times (t_1_ + t_2_) was about 12.5 s, and the presence of PA was reduced (t_1_ + t_2_) by 26.2 s in the sample WAUPM-3. Therefore, PA played a significant role in flame-retardancy in the combustion process of PC/ABS.

In addition, the fire growth index (FGI) was introduced to evaluate the impact of the functionalized MOF coating system on the fire safety performance of PC/ABS. The smaller the value, the less time was required for the material to reach the intense burning state, and the lower the fire risk. As shown in Figure 6b, the FGI value of the untreated PC/ABS was 1.75, while it dropped to 1.07 after coating with MWCNT@UiO-6. The FGI was only 0.38 after the introduction of PA. Therefore, the fire safety performance of PC/ABS was significantly improved by coating with PA@MWCNT@UiO-66.

**Figure 7:**
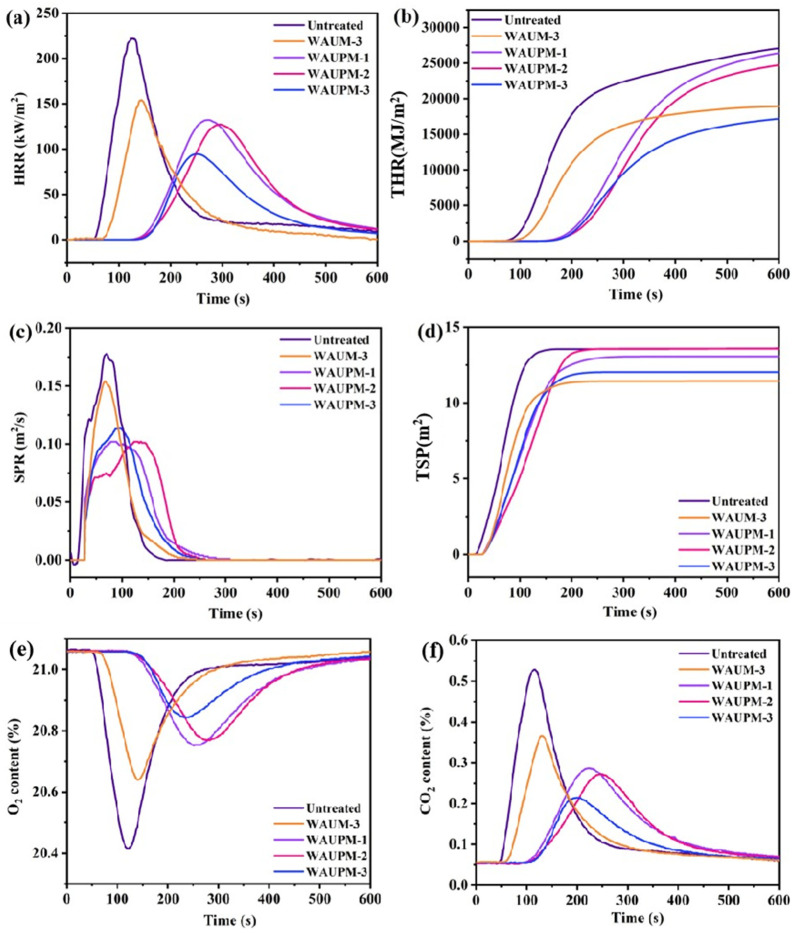
Curves for heat release rate (HPR) (**a**), total heat release (THR) (**b**), smoke production rate (SPR) (**c**), total smoke production (TSR) (**d**), CO_2_ (**e**), and O_2_ (**f**) contents of untreated, WAUM-3, WAUPM-1, WAUPM-2, and WAUPM-3 samples.

Smoke suppression performance is an important factor for the evaluation of the fire resistance of materials. CCT was used to simulate the real fire environment and evaluate the combustion behavior of materials in terms of heat, smoke, CO_2_ release, etc. Figure 7 presents the heat release rate (HPR), total heat release (THR), smoke production rate (SPR), total smoke production (TSR), and curves for CO_2_ and O_2_ content of the samples. Figure 7a showed that the HRR value of the WAUM-3 sample was 153.7 kW/m^2^, which decreased by 26.2% compared with the untreated sample. Figure 7b showed that the TRR value decreased by 26.9% compared with the untreated sample, which implied that the MWCNT@UiO-66 coating could effectively inhibit the heat release of PC/ABS. After the introduction of PA, the HRR and TRR values decreased by 57.5% and 33.6%, respectively, compared with the untreated sample (Figure 7a,b). This result indicated that PA could further inhibit the heat release of PC/ABS. In addition, as the thickness of the PA@MWCNT@UiO-66 coating increased from 250 μm to 750 μm, the peak smoke production rate decreased significantly and smoke release had a delayed effect. WAUM-3 and WAUPM-3 emitted similar amounts of smoke (11.44 m^2^ and 12.03 m^2^), but the WUPM-1, -2, and -3 samples extended the smoke release time and increased the escape time. This implied that MOFs played an important role, due to their high specific surface area and highly ordered porous structure. In the early stage of pyrolysis, the organic flammable volatiles released by PC/ABS passed through a complicated path. PA, as a phosphate, easily decomposes in a degraded polymer matrix to generate phosphoric acid. Phosphoric acid promotes surface cation crosslinking and carbonization, acting as an insulation barrier in the carbonization layer on the polymer surface to suppress thermal feedback from the combustion zone and act as a flame retardant [24]. Therefore, the coating with MOFs resulted in delayed smoke release. As the combustion intensified, PA produced meta-phosphate, polyphosphate, and other compounds, which could undergo dehydration with the hydroxyl group from PC/ABS pyrolysis and promote the formation of a carbon layer. Thus, the cross-linking of PC/ABS with the pyrolysis products of PA@MWCNT@UiO-66 promoted the carbonization process and improved the flame-retardancy. Figure 7e,f showed the changes in CO_2_ and O_2_ contents during the cone calorimetry test. Compared with the untreated sample, the consumption of O_2_ and generation of CO_2_ were reduced and delayed significantly in WAUM-3. Particularly, the time was delayed by more than two times upon the addition of PA in the case of WAUPM. As the coating thickness increased from 250 μm to 750 μm, the overall O_2_ consumption and CO_2_ generation of the material continued to decrease. This result further showed that the thermal oxygen stability of flame-retardant materials increased after the coating of an MOFs-based flame retardant onto PC/ABS materials. It also indicated that the MOFs-based flame-retardant system reduced the reactivity with oxygen by inhibiting the contact between the matrix and O_2_. Thus, the MOFs-based flame-retardant coating inhibited the violent combustion of PC/ABS.

### 3.4. Mode of Action of Flame Retardant

**Figure 8:**
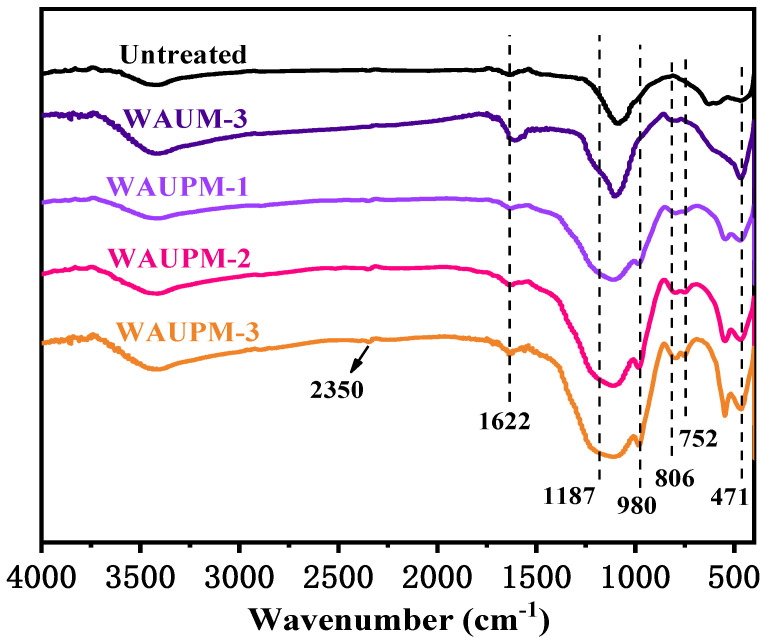
FT-IR spectra of carbon residues after the cone calorimetry test.

In order to understand the mode of action of MOFs-based flame-retardant coatings, the carbon residues were carefully characterized by FT-IR spectroscopy, SEM, and Raman spectroscopy. Figure 8 presents the FT-IR spectra of carbon slags after the cone calorimetry test. WAUM-3 showed weak absorption peaks at 806 cm^−1^ and 752 cm^−1^, ascribed to the out-of-plane deformation vibrations of the benzene ring =C-H, and para-substituted and mono-substituted benzene ring, respectively. The peaks at 1622 cm^−1^ and 471 cm^−1^ corresponded to the aromatic structure and Zr-O stretching vibrations, respectively. This result indicated that zirconia was produced after the combustion of WAUM-3 and that the aromatic structures of UiO-66 promoted the formation of carbon layers. Furthermore, the WAUPM coated samples showed P-O-C stretching vibrations at 980 cm^−1^ and peaks at 1187 cm^−1^ and 1092 cm^−1^ for P=O absorption and Si-O-Si absorption, respectively. This suggested that PA was decomposed during the combustion process to phosphoric acid, meta-phosphate, and other compounds. These compounds were esterified with the pyrolysis products of PC/ABS containing hydroxyl groups. Moreover, the presence of nano silica and carbon nanotubes promoted the formation of dense and continuous coke layers. Hence, as the coating thickness increased, the intensities of both P-O-C and Zr-O peaks in the carbon slag increased, as shown in Figure 8 for WAUPM-1, WAUPM-2, and WAUPM-3. Zirconia produced by the decomposition of zirconium organic framework also served as an efficient thermal barrier when mixed with the carbon layer. Thus, the product improved the thermal stability and thermal shielding property of the carbon layer.

**Figure 9:**
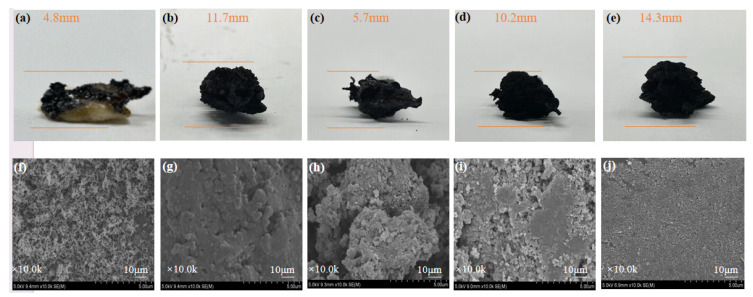
Macroscopic images and SEM images of carbon residues after vertical combustion test: (**a**,**f**) Untreated; (**b**,**g**) WAUM-3; (**c**,**h**) WAUPM-1; (**d**,**i**) WAUPM-2; and (**e**,**j**) WAUM-3.

The residual amounts and the macro- and micro-morphologies of the carbon layers after polymer combustion also reflected the carbonization process. The morphology of the carbon slag in the condensation stage was investigated carefully. From a macro perspective, Figure 9a–d showed that the untreated PC/ABS generated the least amount of carbon, because most of the elements were released into the atmosphere in the form of smoke during combustion. In contrast, the amounts of carbon residues were much higher for WAUM-3 and WAUPM-3. The expanded size of carbon increased as the coating thickness increased for WAUPM-1, WAUPM-2, and WAUPM-3. The morphology of the carbon layer was further studied by SEM. The untreated PC/ABS showed many pores and the carbon layer was relatively loose (Figure 9f). The quality of the carbon layer improved in WAUM-3, but still showed a porous structure with traces of incomplete combustion (Figure 9f). However, the carbon layer of WAUPM-3 showed a smoother surface (Figure 9j). This implied that the carbon layer acted as a physical barrier against heat and inhibited the transfer of oxygen and materials between the condensed and gas phases. Thus, the MOFs-based flame-retardant coating effectively inhibited the thermal degradation of the underlying PC/ABS.

**Figure 10:**
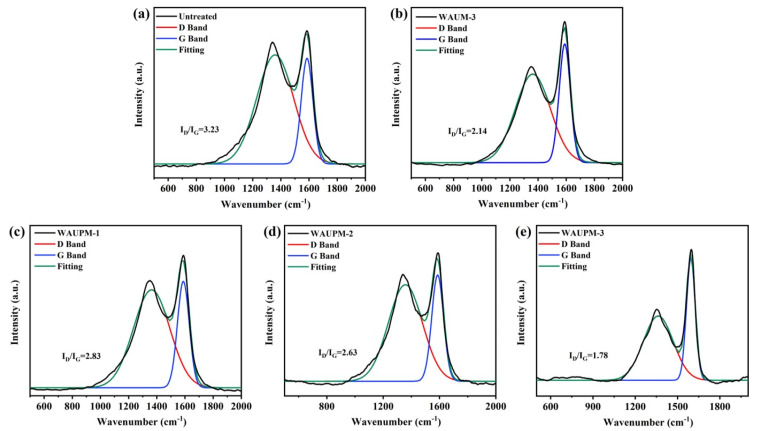
Raman spectra of carbon residues from untreated PC/ABS (**a**), WAUM-3 (**b**), WAUPM-1 (**c**), WAUPM-2 (**d**) WAUM-3, and (**e**) coated PC/ABS.

Raman spectroscopy was employed to further characterize the degree of graphitization. Figure 10 presents the Raman spectra of samples. Peak D and peak G at about 1350 cm^−1^ and 1600 cm^−1^ corresponded to the peaks of amorphous carbon and graphitized carbon, respectively. The higher the degree of graphitization, the better the effect of heat insulation and oxygen isolation. As shown in Figure 10, as the thickness of the coating increased in WAUPM-1, WAUPM-2, and WAUPM-3, the I_D_/I_G_ value decreased. Finally, the carbon residue of WAUPM-3 had the lowest I_D_/I_G_ value. Compared with untreated PC/ABS and WAUM-3, the I_D_/I_G_ value was also much smaller for WAUPM-3. These results indicated that WAUPM-3 had the highest degree of graphitization after carbon combustion, which displayed a good barrier effect and effectively inhibited the thermal degradation of the underlying PC/ABS. This observation was consistent with the SEM results.

**Figure 11:**
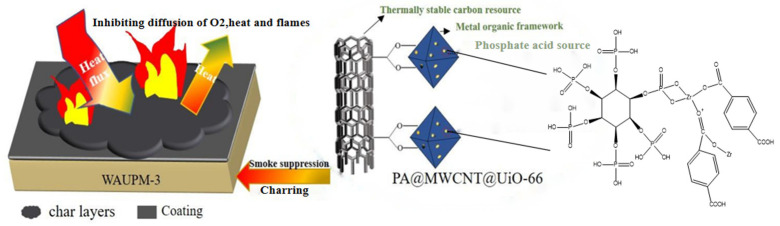
Illustration of the mode of action of fire-retardancy of the MOFs-based flame-retardant coating.

Finally, based on the above analysis, the possible mode of action of the flame-retardant WAUPM-3 is described in Figure 11. When an external heat source or flame was applied, the MOFs-based coating acted as a thermal barrier due to the better thermal stability of PA@MWCNT@UiO-66. This prevented the flame from coming into direct contact with the PC/ABS substrate. In the second stage, when PC/ABS combustion was caused by cracking of the coating due to thermal expansion, the porous structure of MOFs delayed the partial release of smoke. This was because the porous structure of MOFs played a role of catalytic carbonization, forming dense coke that covered the surface of the material. This layer acted as an insulation barrier to inhibit heat transfer from the combustion zone and decrease the rate of formation of volatile fuel fragments. Moreover, MWCNT played another role of promoting the construction of a carbon cross-linking network. In general, the coatings comprised of a phosphorus-carbon-zirconium flame-retardant system endowed PC/ABS with excellent flame-retardancy.

## 4. Conclusions

A new kind of MOFs-based flame retardant was prepared by loading phytic acid (PA) and multi-walled carbon nanotubes (MWCNT) onto UiO-66 by a solvothermal method. It was further applied as a flame-retardant surface coating onto PC/ABS. The results showed that PA improved the compatibility between UiO-66 and the acrylic resin and also helped to maximize the interfacial bonding between the adhesive layer and PC/ABS substrate and improve the wrapping property. When the thickness of the coating was 750 μm, the thermal barrier effect was significant and the LOI value of the material was 27.5%. The cone calorimetry test showed that the heat release and smoke release of the material had significant hysteresis and inhibition effects. Raman spectral analysis showed that a high-quality carbon layer was constructed during the combustion process, which effectively shielded the heat and oxygen transfer to the internal matrix. The phosphorus, carbon, and zirconium elements of PA, MWCNT, and UiO-66 contained in the coating showed flame-retardancy in the condensed phase. The hexagonal structure and thermal stability of MWCNT promoted the formation of a cross-linked network in the carbon layer. UiO-66 was involved in char formation and the tunnel effect during the early and middle stages of pyrolysis to provide the flame-retardant effect. In addition, the flame-retardant coating did not affect the mechanical properties of PC/ABS as in the case of physical filling, and the brushing was easy to repeat.

## Figures and Tables

**Table 1:** Composition of aqueous flame retardants.

Sample	Solid Content (wt%)	Water-Based Acrylic Adhesive (g)	UiO-66 (g)	MWCNT @UiO-66 (g)	PA@MWCNT @UiO-66 (g)	SiO_2_ (g)	H_2_O (g)
WA	41.9	2	0	0	0	0	0.34
WAU	41.9	2	1	0	0	0.2	2
WAUM	41.9	2	0	1	0	0.2	2
WAUPM	41.9	2	0	0	1	0.2	2

**Table 2:** Thickness of flame-retardant coating on PC/ABS.

Sample *	WAU	WAUM	WAUPM	Coating Thickness (μm)
Untreated	×	×	×	0
WAU-3	√	×	×	750
WAUM-3	×	√	×	750
WAUPM-1	×	×	√	250
WAUPM-2	×	×	√	500
WAUPM-3	×	×	√	750

* × represents not using this sample for coating and √ represents using this sample for coating.

## Data Availability

Data are contained within the article.

## Supplementary Materials

The following supporting information can be downloaded at: https://www.mdpi.com/article/10.3390/polym16020275/s1, Figure S1: TGA (a) and DTG (b) curves of UiO-66@PA@MWCNT, UiO-66 and MWCNT-COOH; Figure S2: SEM images of (a) UiO-66; (b) MWCNT-COOH; (c) UiO-66@PA@MWCNT, and elemental distribution (d1–d4) of UiO-66@PA@MWCNT; Table S1: TGA data of samples for WAUPM, WAUM, WAU and WA; Table S2: Results of flame retardant coated PC/ABS for LOI and vertical burning test; Table S3: Results of flame retardant coated PC/ABS for Cone Calorimetry Test.
