# Supplementary material for: Development of a UiO-66 Based Waterborne Flame-Retardant Coating for PC/ABS Material
Source: Polymers (Basel). 2024 Jan 19;16(2):275. doi: 10.3390/polym16020275 (PMC10821372; doi:10.3390/polym16020275)
Supplement: Supplementary file 1 [file polymers-16-00275-s001.zip › polymers-2769604-supplementary.pdf]

Supplementary materials

Table S1. TGA data of samples for WAUPM, WAUM, WAU and WA

	$T_{-5\%}$ (℃)	T_{MAX1} (℃)	Max1 lose rate (%/℃)	T_{MAX2} (℃)	Max2 lose rate (%/℃)	Residues at 800℃ (%)
WA	367.4	433.1	3．41	-	-	1.5
WAU	241.3	427.1	1.07	548.6	0.29	29.9
WAUM	234.6	430.5	1.30	552.7	0.27	30.9
WAUPM	195.9	293.8	0.15	415.1	1.04	38.8

Table S2. Results of flame retardant coated PC/ABS for LOI and vertical burning test

Sample	LOI/%	UL-94	t_1/s	t_2/s	Dripping
Untreated	21.2	NR	＞60	＞60	YES
WAU-3	23.4	NR	＞60	＞60	YES
WAUM-3	26.1	V-1	17.4	21.3	NO
WAUPM-1	22.9	NR	＞60	＞60	YES
WAUPM-2	24.8	V-2	19.6	25.7	YES
WAUPM-3	27.5	V-0	1.7	10.8	NO

Table S3. Results of flame retardant coated PC/ABS for Cone Calorimetry Test

Parameters	Untreated	WAUM-3	WAUPM-1	WAUPM-2	WAUPM-3
PHRR(kW/m^2)	221.9	153.7	132.1	127.2	94.4
t-PHPR(s)	127	143	272	296	249
TRR(kW/m^2)	25870	18913	26383	24693	17167
TSR(m^2/m^2)	13.58	11.44	13.06	13.58	12.03
FGI	1.75	1.07	0.49	0.43	0.38

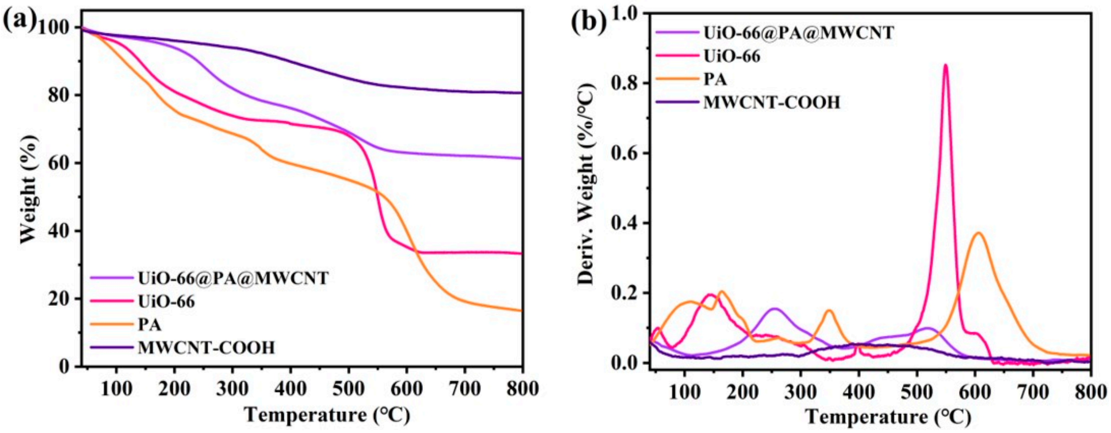

Fig S1. TGA(a) and DTG(b) curves of UiO-66@PA@MWCNT, UiO-66 and MWCNT-COOH

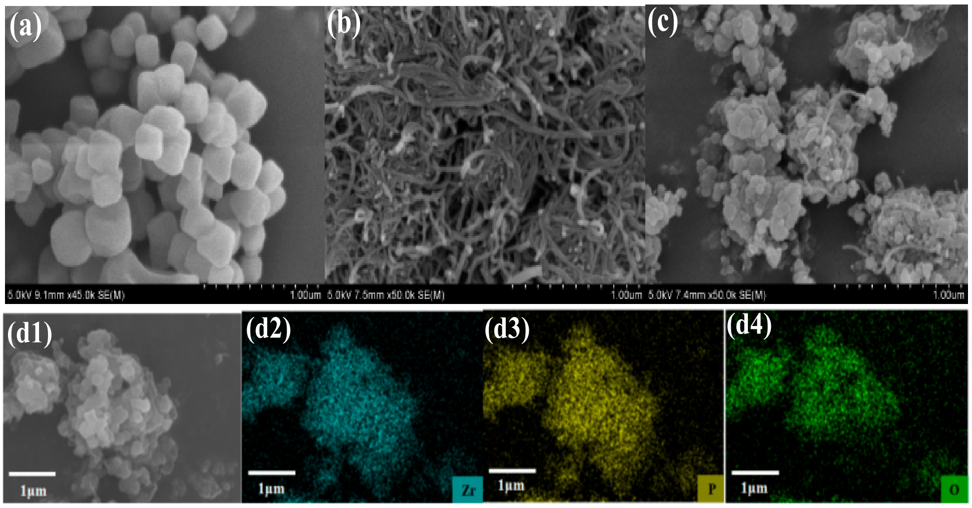

Fig.S2.SEM images of (a) UiO-66; (b) MWCNT-COOH; (c) UiO-66@PA@MWCNT, and elemental distribution (d1-d4) of UiO-66@PA@MWCNT
